# Perceived health literacy and COVID-19 vaccine acceptance among Chinese college students: A mediation analysis

**DOI:** 10.1371/journal.pone.0273285

**Published:** 2022-09-02

**Authors:** Fangfang Jiang, Yang Zhao, Jianling Bai, Xueying Yang, Jiajia Zhang, Danhua Lin, Xiaoming Li

**Affiliations:** 1 Department of Mathematical Sciences, School of Social Sciences, University of Southampton, Southampton, Hampshire, United Kingdom; 2 Department of Epidemiology and Biostatistics, School of Public Health, Nanjing Medical University, Nanjing, Jiangsu, China; 3 South Carolina SmartState Center for Healthcare Quality, Arnold School of Public Health, University of South Carolina, Columbia, South Carolina, United States of America; 4 Department of Health Promotion, Education and Behavior, Arnold School of Public Health, University of South Carolina, Columbia, South Carolina, United States of America; 5 Department of Epidemiology and Biostatistics, Arnold School of Public Health, University of South Carolina, Columbia, South Carolina, United States of America; 6 Institute of Developmental Psychology, Faculty of Psychology, Beijing Normal University, Beijing, China; The University of the West Indies, TRINIDAD AND TOBAGO

## Abstract

**Background:**

Although COVID-19 vaccines hold the potential to dramatically alter the COVID-19 pandemic, vaccine hesitancy remains a serious threat to the management and control of COVID-19 infections. Vaccination of young adults plays a crucial role in achieving herd immunity. However, the determinants of COVID-19 vaccine acceptance among the youth in China have not been fully explored. Our study aims to investigate the direct and indirect effects of perceived health literacy on COVID-19 vaccine acceptance.

**Methods:**

This survey was conducted among Chinese college students during September and October, 2020. COVID-19 vaccine acceptance was defined as the likelihood that participants would get a COVID-19 vaccine. A mediation analysis was employed to explore the direct and indirect effects of perceived health literacy on COVID-19 vaccine acceptance.

**Results:**

A total of 2,587 college students were included in our study. The results of the survey revealed that the majority (80.40%) of the participants expressed high COVID-19 vaccine acceptance. After controlling for demographic characteristics, the relationship between perceived health literacy and COVID-19 vaccine acceptance was mediated by positive attitudes toward general vaccination (std.β = 0.004, p = 0.037) and self-efficacy of COVID-19 vaccine (std.β = 0.053, p < 0.001).

**Conclusions:**

The findings suggest that interventions targeting health literacy to promote COVID-19 vaccination coverage might consider placing greater emphasis on enhancing the positive attitude towards and self-efficacy of vaccines among youth.

## Background

The coronavirus disease 2019 (COVID-19), which is caused by severe acute respiratory syndrome (SARS)-CoV-2, has posed a great threat to the population health worldwide. Started from Wuhan city of China at the end of December 2019, SARS-CoV-2 infection is characterized by a wide range of symptoms, varying from mild to severe respiratory distress syndrome [1]. Transmission of SARS-CoV-2 can occur through human-to-human transmissions such as direct, contact transmission and airborne transmission through aerosols [2, 3]. As of July 8, 2022, more than 551 million confirmed COVID-19 cases including 6.34 million deaths emerged around the world [4]. COVID-19 is detected mainly by RT-PCR using nasal swabs or pharyngeal swabs. To date, there is no single worldwide approved therapeutic options for COVID-19 patients, although retrospective studies reported the efficacy of remdesivir and lopinavir/ritonavir in reducing viral load, convalescent plasma in boosting immunity, and statin use in reducing disease severity [5–7].

Given the rapid transmission and unavailable treatment of COVID-19, effective and durable SARS-CoV-2 vaccines hold the potential to dramatically alter the COVID-19 pandemic [8, 9]. Remarkable achievement of COVID-19 vaccine development was observed in large, randomized-controlled trials [10, 11], where several vaccines were found to be safe and efficacious in preventing symptomatic COVID-19, such as mRNA vaccines (e.g., BNT162b2 [Pfizer/BioNTech] and mRNA-1273 [Moderna]], adenovirus vector-based vaccines (e.g., Ad26.COV2.S [J&J/Janssen]), and inactivated vaccines (e.g., CoronaVac [Sinovac]) [12]. It is reported that the inactivated COVID-19 vaccines most widely used in China (e.g., BBIBP-CorV and CoronaVac) have a less effectiveness (66%-78%) [13, 14] than the mRNA vaccines (BNT162b2 and mRNA-1273, >90%) used in Western countries [15, 16]. Despite the proven effectiveness of COVID-19 vaccines in preventing severe COVID-19 outcomes, the global vaccination coverage remains far below expectations [17]. Geographic disparities in vaccination coverage exist around the world. For example, only less than half of the population are fully vaccinated in underdeveloped countries (32.2% and 40.7% in South Africa and Jordan), while such rate are much higher in more economically developed areas (67% in the United States, 74.7% in the United Kingdom and 88.5% in China) [18]. The uneven vaccination status is a serious impediment to ending the pandemic. With the emergence of the new variants—Delta and Omicron variants—global vaccination has become imperative.

As one of the top ten global health threats, vaccine hesitancy is a long-term obstacle to controlling infectious disease pandemics [19]. Vaccine hesitancy is defined as the delay in acceptance or refusal of vaccination despite the availability of vaccination services [20]. An online global survey in 2020 showed that around 27% of people expressed COVID-19 vaccine hesitancy, highest in France (46%), followed by the United States (36%) and Spain (36%) [21]. At the beginning of this survey, the majority of respondents were willing to be vaccinated, but their vaccine acceptance dropped sharply within two months (12% in China, 9% in Australia and 8% in Spain). A similar trend was also observed by Wang et al. where Chinese people became progressively more hesitant to get vaccinated [22]. In India and Germany, nearly 40% of the population expressed COVID-19 vaccine hesitancy [23, 24]. Since COVID-19 vaccination is a significant cost-effective intervention, improving the global COVID-19 vaccine acceptance is critical to the management of COVID-19 [25].

Previous studies have found that knowledge about vaccination, trust in efficacy and safety of vaccines and trust in health professionals were significantly related to the attitude towards general vaccines [26, 27]. During the outbreak of COVID-19, there is a growing body of literature on COVID-19 vaccine acceptance. Sex, age, and education in many regions were associated with lower COVID-19 vaccine acceptance [23, 28–30]. Lack of knowledge about COVID-19 vaccines was found to decrease the vaccine acceptance in India, while preference for natural immunity acted as a barrier in the UK [24, 29]. Besides, previous studies provided important insights into the crucial role of distrust in efficacy and safety of vaccines, existing co-morbidities, and knowledge about COVID-19 vaccines on COVID-19 vaccine acceptance [24, 29, 31–33]. Apart from these factors, health engagement, vaccination history and health literacy were also significant factors associated with COVID-19 vaccine acceptance [31, 32, 34]. Despite the fact that young adults tend to have higher perceived health literacy than the older adult [35], young adults are suspected to be the most hesitant group to receive the COVID-19 vaccine [36].

**Fig 1:**
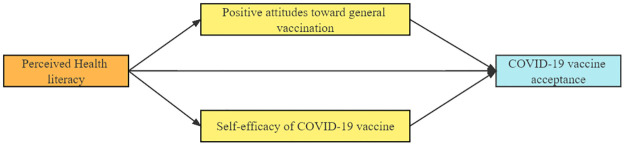
Theoretical mediation model.

Given the public health significance, our study aims to investigate the effect of perceived health literacy on COVID-19 vaccine acceptance as well as the roles of positive attitudes toward general vaccination and self-efficacy of COVID-19 vaccine in mediating the association among Chinese college students. The mediation model was developed and displayed in Fig 1. Several model hypotheses were proposed as follows: (1) perceived health literacy would be directly associated with COVID-19 vaccine acceptance; (2) positive attitudes toward general vaccination and self-efficacy of COVID-19 vaccine would have a direct association with COVID-19 vaccine acceptance; and (3) positive attitudes toward general vaccination and self-efficacy of COVID-19 vaccine would mediate the association between perceived health literacy and COVID-19 vaccine acceptance.

## Method

### Study design and participants

**Fig 2:**
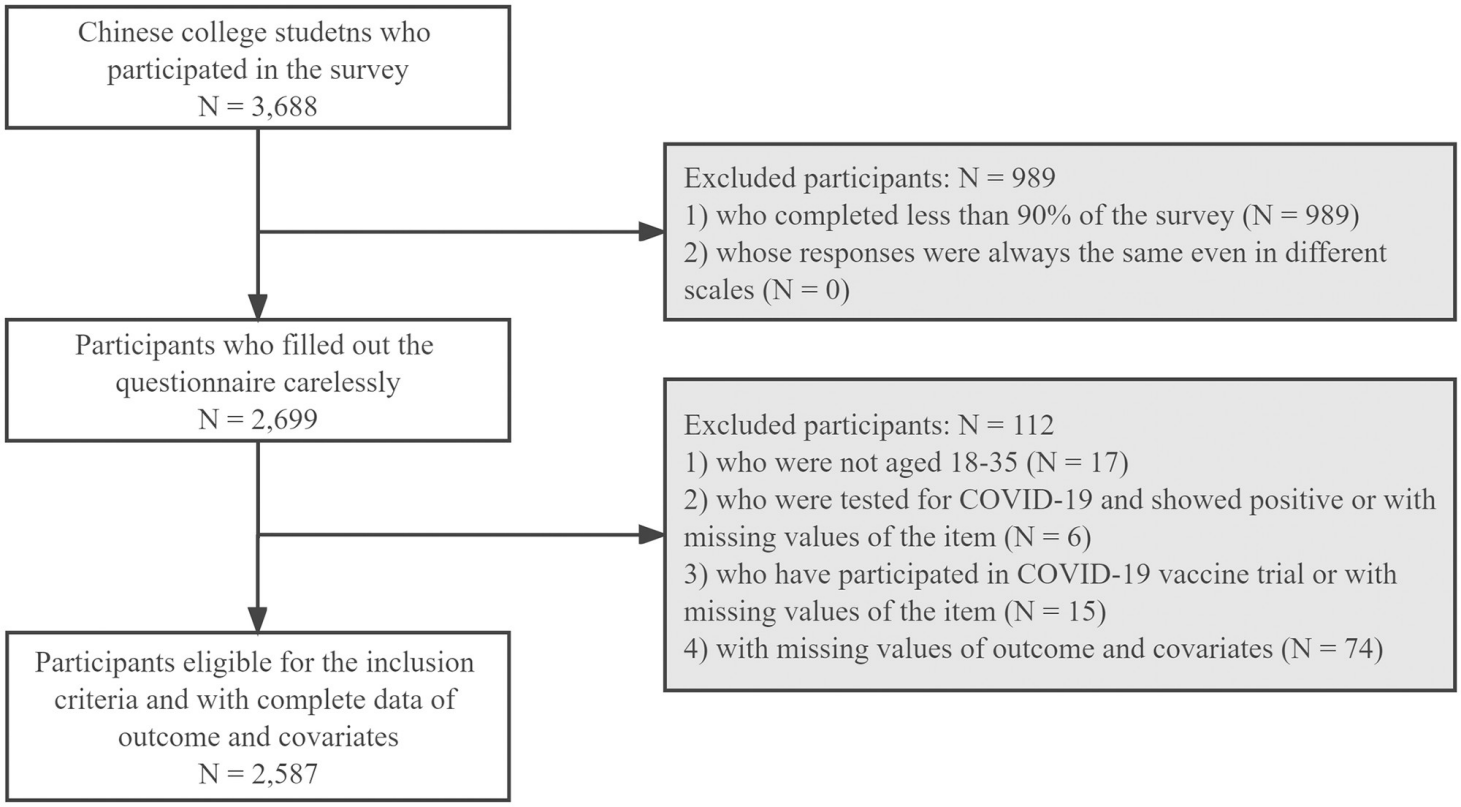
Flow chart population included in our analysis (N = 2,587).

Between September and October 2020, we conducted an online survey among college students in 73 universities across China. The participants were recruited through a convenience sampling method with inclusion criteria: (1) being 18–35 years old; and (2) being college students who were currently enrolled in universities in China. Exclusion criteria were students (1) who filled out the questionnaire carelessly (e.g., participants whose responses always were the same in different scales or who completed less than the 90% of the survey); (2) who were tested for COVID-19 and showed positive; (3) who have participated in COVID-19 vaccine trial; and (4) with missing values of outcome and covariates. This anonymous questionnaire survey was developed using SO JUMP system technology, a well-known Chinese online survey platform similar to Amazon Mechanical Turk in health surveys [37]. College students were invited to participate in the survey via email. The online informed consent was obtained prior to the survey. Participants were allowed to share the link with other students. More details about the data collection can be found in the previous study [38]. The final sample comprised of 2,587 Chinese college students (Fig 2).

### Measures

#### Demographics

Participants were asked to report their sociodemographic information, including gender (female, male, or others), age, school year, housing arrangement (on campus vs off campus), and annual family income (<$1,500, $1,500-$7,500, $7,500-$15,000, >$15,000). Due to the small number of participants (< 5%) in certain categories, we dichotomized gender (0 = female, 1 = male) and school year (0 = undergraduate, 1 = graduate) for data analysis.

#### Perceived health literacy

Perceived health literacy was assessed using an 11-item scale. The items described individuals’ understanding about health care (e.g., “Understand what your doctor says to you”), health promotion (e.g., “Understand information on food packaging”), and disease prevention (e.g., “Understand why you need vaccinations”), all of which were adapted from the 47-item European perceived health literacy Survey Questionnaire (HLS-EU-Q47) [39]. Participants responded items on a 4-item scale (1 = Very easy, 2 = Fairly easy, 3 = Fairly difficult, 4 = Very difficult). In the current study, the score of each item was coded reversely. A sum score was calculated from 11 items, with a higher sum score indicating a higher level of perceived health literacy. The internal consistency was excellent (Cronbach’s α = 0.898).

#### Positive attitudes toward general vaccination

Four items were measured to evaluate participants’ positive attitudes toward general vaccination. Participants reported their trust in safety of COVID-19 vaccines, such as “Vaccines are safe” and “Vaccines have side effects”. The responses to each item ranged from 1 (Strongly disagree) to 7 (Strongly agree) on a seven-point Likert scale. The score of three items was reversely coded for data analysis. A sum score was generated, with a higher sum score indicating a higher level of positive attitudes toward general vaccination. The Cronbach’s α was 0.465.

#### Self-efficacy about COVID-19 vaccine

A 4-item, self-developed scale was used to assess participants’ confidences in their capabilities to perform the recommended behavior (e.g., “Even if nobody I know is willing to take the vaccine, I will take it”). Each question was rated using a five-point Likert scale (1 = Strongly disagree, 2 = Slightly disagree, 3 = Not sure, 4 = Slightly agree, 5 = Strongly agree). A sum score was created with a higher sum score indicating a greater self-efficacy of COVID-19 vaccination. The Cronbach’s α of this scale was 0.820.

#### COVID-19 vaccine acceptance

One question was used to evaluate participants’ likelihood to take a COVID-19 vaccine in the future (i.e., “How likely will you take a COVID-19 vaccine when it is available?”). Participants answered this question on a 5-point Likert scale (1 = Definitely not take it, 2 = Likely not take it, 3 = I don’t know yet, 4 = Likely take it, 5 = Definitely take it). A higher score represented a greater level of vaccine acceptance.

### Statistical analysis

First, multiple imputations were conducted to handle the missing values in data. Descriptive analyses were performed to describe the sociodemographic characteristics (i.e., age, gender, school year, major, housing arrangement, shared accommodation, and annual family income) and COVID-19 vaccine acceptance. Continuous variables were presented using mean (SD) and median (first quartile, second quartile). Frequency (ratio) was utilized to describe the characteristics of categorical variables. Second, correlation analyses were performed to investigate the bivariate relationship between variables of interest. Third, a mediation model analysis was conducted to examine whether self-efficacy of COVID-19 vaccine and positive attitudes toward general vaccination could mediate the association between perceived health literacy and COVID-19 vaccine acceptance, controlling for potential confounders (i.e., age, gender, housing arrangement). The standardized direct, indirect, total effects and their 95% confidence intervals (CIs) for the mediation model were then estimated using 5,000 bootstrap samples.

To evaluate the goodness of fit of this mediation model, we employed a series of indices: a χ^2^/df ratio < 5 is considered an index of good fit; Tucker-Lewis Index (TLI) is considered acceptable above 0.90 (or excellent fit if 0.95 or above); Root Mean Square Error of Approximation (RMSEA) and 90% CI is considered acceptable when below 0.08 (or excellent fit if 0.05 or below); Standardized Root Mean Square Residual (SRMR) is considered acceptable below 0.08 (or excellent fit if 0.05 or below) as well; and finally, Comparative Fit Index (CFI) is considered acceptable above 0.90 (or excellent fit if 0.95 or above). All data management and analyses were conducted using mice and lavaan packages in the R 4.0.3 version.

### Ethics statement

The ethical approval for the study was obtained from the Institutional Review Board of the Faculty of Psychology, Beijing Normal University (BNU). An online informed consent from all individual participants was obtained before the survey.

## Results

### Sample characteristics

The demographic characteristics of the sample were presented in Table 1. Among the 2,587 participants, the mean age was 20.42 years old (SD = 1.95). Most participants were female (62.78%), lived on campus (96.25%), and shared accommodation with others (95.21%). More than half of the participants were undergraduates (6.88% freshmen, 46.50% sophomore, 25.13% junior, and 9.93% senior). More than 60% of participants reported an annual family income of $1,500 (equivalent to 10,000 Chinese currency Yuan at the time of survey) or more. About 80.4% of participants reported that they would definitely or likely take a COVID-19 vaccine, 9.12% were not sure about the COVID-19 vaccination, and 10.48% expressed that they would definitely not or likely not take a COVID-19 vaccine.

**Table 1:** Sample characteristics among Chinese college students.

Variable	Overall (N, %)
	N = 2,587
**Age (Year)**	
Mean(SD)	20.42(1.95)
Median(Q1:Q3)	20(19:21)
**Gender**	
Female	1624(62.78)
Male	957(36.99)
Other gender	6(0.23)
**School year**	
Freshman	178(6.88)
Sophomore	1203(46.50)
Junior	650(25.13)
Senior	257(9.93)
Master’s student, first year	165(6.38)
Master’s student, second year or above	99(3.83)
Doctoral student, first year	11(0.43)
Doctoral student, second year or above	24(0.93)
**Housing arrangement**	
On campus	2490(96.25)
Off campus	97(3.75)
**Shared accommodation**	
No	124(4.79)
Yes	2463(95.21)
**Annual family income**	
< $1,500	915(35.37)
$1,500 to $7,500	1156(44.68)
$7,500 to $15,000	283(10.94)
> $15,000	233(9.01)
**COVID-19 vaccine acceptance**	
Definitely take it	569(21.99)
Likely take it	1511(58.41)
Likely not take it	255(9.86)
Definitely not take it	16(0.62)
I don’t know yet	236(9.12)

### Correlation analyses

Table 2 showed the mean scores and standard deviations of the variables, as well as the correlation matrix of all the variables in the mediation model. COVID-19 vaccine acceptance was positively associated with perceived health literacy (r = 0.09, p < .001), positive attitudes toward general vaccination (r = 0.09, p < .001), and self-efficacy of COVID-19 vaccine (r = 0.36, p < .001). Perceived health literacy was positively and significantly correlated with positive attitudes toward general vaccination (r = 0.07, p < .001) and self-efficacy of COVID-19 vaccine (r = 0.15, p < .001). A higher level of positive attitudes toward general vaccination was positively associated with a greater self-efficacy of COVID-19 vaccine (r = 0.09, p < .001).

**Table 2:** Correlation matrix of measured variables.

	Variable	Mean±SD	1	2	3	4
**1**	**COVID-19 vaccine acceptance**	3.91±0.87	1			
**2**	**Perceived health literacy**	33.54±4.69	0.09***	1		
**3**	**Positive attitudes toward general vaccination**	15.85±3.04	0.09***	0.07***	1	
**4**	**Self-efficacy of COVID-19 vaccine**	13.35±2.68	0.36***	0.15***	0.09***	1

SD, standard deviation.

*** p values were less than 0.001.

### Mediation analysis

Perceived health literacy had a significantly positive effect (std.β = 0.09, p < .001) on COVID-19 vaccine acceptance in the absence of mediators. After controlling for covariates (i.e., gender, age, housing arrangement), the final model showed an excellent model fit: χ2/df = 2.913; TLI = 0.926; RMSEA = 0.027 (LO90 = 0.015; HI90 = 0.040); SRMR = 0.018; and CFI = 0.963. Overall, the model was acceptable.

**Fig 3:**
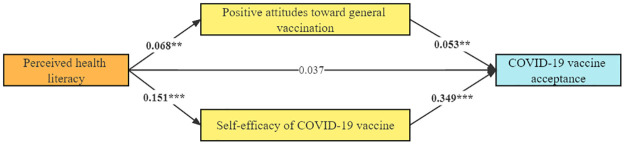
The mediation model of perceived health literacy on COVID-19 vaccine acceptance. The numbers on arrows represent standardized coefficients. P-values marked with bold indicate statistically significant p-values. ** p < .01; *** p < .001.

A multiple mediation model with two mediators and a direct path was established and showed in Fig 3. Specifically, there was a significant, moderate positive relationship between self-efficacy of COVID-19 vaccine and COVID-19 vaccine acceptance (std.β = 0.349, p < 0.001). Moreover, there was also a significant, positive relationship between positive attitudes toward general vaccination and COVID-19 vaccine acceptance (std.β = 0.053, p < 0.01). However, in this mediation analysis, there was no significant, direct relationship between perceived health literacy and COVID-19 vaccine acceptance (p > .05) after considering the indirect effects.

As shown in Table 3, the effect of perceived health literacy on COVID-19 vaccine acceptance was completely mediated by positive attitudes toward general vaccination and self-efficacy of COVID-19 vaccine, with standardized indirect effects of 0.004 (95% CI = [0.001,0.008]) and 0.053 (95% CI = [0.034,0.073]), respectively. Overall, the indirect effects accounted for most of the total effect of perceived health literacy on COVID-19 vaccine acceptance (60.22%).

**Table 3:** Standardized direct, indirect, and total effects for the mediation model.

Model pathways	Standardized estimated effects	p
	(95% CI ^a^)	
Indirect effects	0.056 (0.037,0.076)	< .001
Perceived health literacy -> Positive attitudes toward general vaccination -> COVID-19 vaccine acceptance	0.004 (0.001,0.008)	0.037
Perceived health literacy -> Self-efficacy of COVID-19 vaccine -> COVID-19 vaccine acceptance	0.053 (0.034,0.073)	< .001
Direct effect	0.037 (-0.005,0.081)	0.093
Total effects ^b^	0.093 (0.048,0.137)	< .001

^a^ Bootstrapped 95% Confidence intervals (N = 5000);

^b^ Total effect = indirect effect + direct effect.

## Discussion

Vaccine hesitancy is a threat to halting the spread of COVID-19 globally. It is necessary to understand Chinese college students’ attitudes towards COVID-19 vaccine to prevent and control the epidemic. This study was conducted in time to investigate the likelihood of college students receiving a COVID-19 vaccine during the COVID-19 recovery period in China. The majority of participants in this study were willing to receive the COVID-19 vaccine, which is consistent with previous findings in China [40]. But the rate of COVID-19 vaccine hesitancy (20%) in this study differs from the findings of western college students. It was slightly higher than that in Italy (13.9%) [41], but much lower than the rates in the United States (47.5%) [42], Egypt (45.7%) [43] and Jordan (39.6%) [44]. These differences may be attributed to social-demographic factors, attitude towards natural immunity, national policy and the level of perceived health literacy in different regions [29, 41, 45].

In this study, respondents generally had high levels of perceived health literacy. However, they usually cannot fully understand the meaning conveyed by doctors and drug leaflets (S1 Table). Our study found that perceived health literacy was positively related to COVID-19 vaccine acceptance in the absence of mediators. In the mediation model, the effect of perceived health literacy on COVID-19 vaccine acceptance was completely mediated by positive attitudes toward general vaccination and self-efficacy of COVID-19 vaccine; and there was no direct relationship between perceived health literacy and COVID-19 vaccine acceptance. In the United States, it was found that perceived health literacy was associated with COVID-19 vaccine acceptance among college students [45]. The main reason might be that while higher levels of perceived health literacy (e.g., understanding recommendations from physicians) are crucial to promoting vaccine acceptance [21], perceived health literacy in this study mainly focused on people’s daily medical cognition and lacked vaccine literacy [35]. Second, the association between perceived health literacy and COVID-19 vaccine acceptance was offset by the influence of positive attitudes toward general vaccination and self-efficacy of COVID-19 vaccine on COVID-19 vaccine acceptance. Overall, perceived health literacy still plays a crucial role on COVID-19 vaccine acceptance. Universities are recommended to incorporate health literacy into the curriculum as part of the university’s health communication strategy to deliver effective health information and help increase COVID-19 vaccine acceptance.

Extant literature showed that students with a higher level of knowledge about COVID-19 vaccine had higher COVID-19 vaccine acceptance [43]. It has been previously observed that students who received vaccines in the past were far more likely to recommend the same vaccine to their friends and family [46]. Distrust in efficacy and safety of COVID-19 vaccine, incorrect COVID-19 vaccine information, and preference for natural immunity always make individuals feel negative about vaccines [29, 43]. Generally, in the current study participants hold high levels of positive attitudes toward general vaccination. But some Chinese college students showed distrust in safety of COVID-19 vaccine. Participants in this survey preferred vaccination to natural immunity compared with western populations [28].

The findings indicated that positive attitudes toward general vaccination would be an important determinant of higher COVID-19 vaccine acceptance. Encouraging colleges to design health education programs and media to provide medical information can lower students’ cognitive bias and distrust [47]. In addition, as a mediator and a predictor, self-efficacy of COVID-19 vaccine have a positive effect on COVID-19 vaccine acceptance, indicating that with more belief in their capabilities in taking a COVID-19 vaccine, there will be a trend that college students are more willing to receive a vaccine. How to reduce college students’ vaccine hesitancy remains an important public health problem. Government supports are important to implement COVID-19 vaccination. In Caribbean regions, the COVID-19 vaccine coverage has improved a lot under the support of local health facilities and WHO [48]. Considering the complexity of vaccine hesitancy, the government needs to develop specialized strategies for different populations [49].

This study has some limitations. First, most scales in our survey were not validated and some of them showed low internal reliability (e.g., positive attitude toward general vaccine). Second, a convenient sampling method was used in this study, which might not be representative of all the college students in China. Third, this study is a cross-sectional survey and causal relationships could not be inferred. Fourth, the effects of unknown confounders cannot be adjusted in the model. Fifth, although the path model was acceptable, as some effect sizes were small, the regression estimates of indirect, direct, and total effects should be interpreted cautiously.

## Conclusions

This study provides preliminary findings regarding the association of perceived health literacy with COVID-19 vaccine acceptance and how this association was mediated by positive attitudes toward general vaccination and self-efficacy of COVID-19 vaccine. The government, health authority decision-makers, medical experts, and universities in China need to work together and put more efforts to build vaccine confidence in general, and subsequently improve the COVID-19 vaccine acceptance. Our findings also highlighted the importance of increasing self-efficacy of COVID-19 vaccine. Although a high vaccine acceptance rate has been observed, there are still some barriers in the process of moving from the vaccination intention to real uptake behavior. Since a vaccination program has been carried out in China and a large number of Chinese residences have taken a COVID-19 vaccine, actual vaccine acceptance needs to be explored in the future to achieve herd immunity.

## Supporting information

S1 Table Descriptive statistics and composite reliability of survey measures. (DOCX)

S1 File (DOCX)

## References

[1] Umakanthan S, Sahu P, Ranade AV, Bukelo MM, Rao JS, Abrahao-Machado LF, et al. Origin, transmission, diagnosis and management of coronavirus disease 2019 (COVID-19). Postgrad Med J. 2020;96: 753–758. doi: 10.1136/postgradmedj-2020-138234 32563999 PMC10016932

[2] Li Q, Guan X, Wu P, Wang X, Zhou L, Tong Y, et al. Early Transmission Dynamics in Wuhan, China, of Novel Coronavirus-Infected Pneumonia. N Engl J Med. 2020;382: 1199–1207. doi: 10.1056/NEJMoa2001316 31995857 PMC7121484

[3] Chen N, Zhou M, Dong X, Qu J, Gong F, Han Y, et al. Epidemiological and clinical characteristics of 99 cases of 2019 novel coronavirus pneumonia in Wuhan, China: a descriptive study. The Lancet. 2020;395: 507–513. doi: 10.1016/S0140-6736(20)30211-7 32007143 PMC7135076

[4] WHO Coronavirus (COVID-19) Dashboard. [cited 20 Jul 2022]. https://covid19.who.int

[5] Abubakar AR, Sani IH, Godman B, Kumar S, Islam S, Jahan I, et al. Systematic Review on the Therapeutic Options for COVID-19: Clinical Evidence of Drug Efficacy and Implications. Infect Drug Resist. 2020;13: 4673–4695. doi: 10.2147/IDR.S289037 33402839 PMC7778508

[6] Zhang X-J, Qin J-J, Cheng X, Shen L, Zhao Y-C, Yuan Y, et al. In-Hospital Use of Statins Is Associated with a Reduced Risk of Mortality among Individuals with COVID-19. Cell Metab. 2020;32: 176–187.e4. doi: 10.1016/j.cmet.2020.06.015 32592657 PMC7311917

[7] Umakanthan S, Senthil S, John S, Madhavan MK, Das J, Patil S, et al. The protective role of statins in COVID-19 patients: a retrospective observational study. Transl Med Commun. 2021;6: 22. doi: 10.1186/s41231-021-00102-4 34604534 PMC8475829

[8] Cohen MS, Corey L. Combination prevention for COVID-19. Science. 2020;368: 551. doi: 10.1126/science.abc5798 32381692

[9] Corey L, Mascola JR, Fauci AS, Collins FS. A strategic approach to COVID-19 vaccine R&D. Science. 2020;368: 948–950. doi: 10.1126/science.abc5312 32393526

[10] Thomas SJ, Moreira ED, Kitchin N, Absalon J, Gurtman A, Lockhart S, et al. Safety and Efficacy of the BNT162b2 mRNA Covid-19 Vaccine through 6 Months. New England Journal of Medicine. 2021;385: 1761–1773. doi: 10.1056/NEJMoa2110345 34525277 PMC8461570

[11] Baden LR, El Sahly HM, Essink B, Kotloff K, Frey S, Novak R, et al. Efficacy and Safety of the mRNA-1273 SARS-CoV-2 Vaccine. New England Journal of Medicine. 2021;384: 403–416. doi: 10.1056/NEJMoa2035389 33378609 PMC7787219

[12] COVID-19 Vaccines with WHO Emergency Use Listing. In: WHO—Prequalification of Medical Products (IVDs, Medicines, Vaccines and Immunization Devices, Vector Control) [Internet]. 3 Nov 2021 [cited 20 Jul 2022]. https://extranet.who.int/pqweb/vaccines/vaccinescovid-19-vaccine-eul-issued

[13] Wang C, Chen L-Y, Lu Q-B, Cui F. Vaccination with the Inactivated Vaccine (Sinopharm BBIBP-CorV) Ensures Protection against SARS-CoV-2 Related Disease. Vaccines (Basel). 2022;10: 920. doi: 10.3390/vaccines10060920 35746530 PMC9227975

[14] Jara A, Undurraga EA, González C, Paredes F, Fontecilla T, Jara G, et al. Effectiveness of an Inactivated SARS-CoV-2 Vaccine in Chile. N Engl J Med. 2021;385: 875–884. doi: 10.1056/NEJMoa2107715 34233097 PMC8279092

[15] Ghazy RM, Ashmawy R, Hamdy NA, Elhadi YAM, Reyad OA, Elmalawany D, et al. Efficacy and Effectiveness of SARS-CoV-2 Vaccines: A Systematic Review and Meta-Analysis. Vaccines. 2022;10: 350. doi: 10.3390/vaccines10030350 35334982 PMC8948677

[16] Francis AI, Ghany S, Gilkes T, Umakanthan S. Review of COVID-19 vaccine subtypes, efficacy and geographical distributions. Postgrad Med J. 2022;98: 389–394. doi: 10.1136/postgradmedj-2021-140654 37066438

[17] COVID-19 vaccines. [cited 20 Jul 2022]. https://www.who.int/emergencies/diseases/novel-coronavirus-2019/covid-19-vaccines

[18] Ritchie H, Mathieu E, Rodés-Guirao L, Appel C, Giattino C, Ortiz-Ospina E, et al. Coronavirus Pandemic (COVID-19). Our World in Data. 2020 [cited 20 Jul 2022]. https://ourworldindata.org/covid-vaccinations

[19] Mitchell C, https://www.facebook.com/pahowho. PAHO/WHO | Ten threats to global health in 2019. In: Pan American Health Organization / World Health Organization [Internet]. 17 Jan 2019 [cited 20 Jul 2022]. https://www3.paho.org/hq/index.php?option=com_content&view=article&id=14916:ten-threats-to-global-health-in-2019&Itemid=135&lang=en

[20] Meeting of the Strategic Advisory Group of Experts on immunization, April 2014 –- conclusions and recommendations. Wkly Epidemiol Rec. 2014;89: 221–236. 24864348

[21] Global attitudes on a COVID-19 vaccine. Ipsos survey for The World Economic Forum. https://www.ipsos.com/sites/default/files/ct/news/documents/2020-11/global-attitudes-on-a-covid-19-vaccine-oct-2020.pdf

[22] Wang J, Lu X, Lai X, Lyu Y, Zhang H, Fenghuang Y, et al. The Changing Acceptance of COVID-19 Vaccination in Different Epidemic Phases in China: A Longitudinal Study. Vaccines. 2021;9: 191. doi: 10.3390/vaccines9030191 33668923 PMC7996493

[23] Umakanthan S, Lawrence S. Predictors of COVID-19 vaccine hesitancy in Germany: a cross-sectional, population-based study. Postgrad Med J. 2022; postgradmedj-2021-141365. doi: 10.1136/postgradmedj-2021-141365 37062994

[24] Umakanthan S, Patil S, Subramaniam N, Sharma R. COVID-19 Vaccine Hesitancy and Resistance in India Explored through a Population-Based Longitudinal Survey. Vaccines. 2021;9: 1064. doi: 10.3390/vaccines9101064 34696172 PMC8537475

[25] Siedner MJ, Alba C, Fitzmaurice KP, Gilbert RF, Scott JA, Shebl FM, et al. Cost-effectiveness of COVID-19 vaccination in low- and middle-income countries. J Infect Dis. 2022; jiac243. doi: 10.1093/infdis/jiac243 35696544 PMC9214172

[26] Larson HJ, Jarrett C, Eckersberger E, Smith DMD, Paterson P. Understanding vaccine hesitancy around vaccines and vaccination from a global perspective: a systematic review of published literature, 2007–2012. Vaccine. 2014;32: 2150–2159. doi: 10.1016/j.vaccine.2014.01.081 24598724

[27] Brown AL, Sperandio M, Turssi CP, Leite RMA, Berton VF, Succi RM, et al. Vaccine confidence and hesitancy in Brazil. Cad Saude Publica. 2018;34: e00011618. doi: 10.1590/0102-311X00011618 30281705

[28] Malik AA, McFadden SM, Elharake J, Omer SB. Determinants of COVID-19 vaccine acceptance in the US. EClinicalMedicine. 2020;26: 100495. doi: 10.1016/j.eclinm.2020.100495 32838242 PMC7423333

[29] Paul E, Steptoe A, Fancourt D. Attitudes towards vaccines and intention to vaccinate against COVID-19: Implications for public health communications. Lancet Reg Health Eur. 2021;1: 100012. doi: 10.1016/j.lanepe.2020.100012 33954296 PMC7834475

[30] Schwarzinger M, Watson V, Arwidson P, Alla F, Luchini S. COVID-19 vaccine hesitancy in a representative working-age population in France: a survey experiment based on vaccine characteristics. The Lancet Public Health. 2021;6: e210–e221. doi: 10.1016/S2468-2667(21)00012-8 33556325 PMC7864787

[31] Chu H, Liu S. Integrating health behavior theories to predict American’s intention to receive a COVID-19 vaccine. Patient Educ Couns. 2021;104: 1878–1886. doi: 10.1016/j.pec.2021.02.031 33632632 PMC7889032

[32] Graffigna G, Palamenghi L, Boccia S, Barello S. Relationship between Citizens’ Health Engagement and Intention to Take the COVID-19 Vaccine in Italy: A Mediation Analysis. Vaccines. 2020;8: 576. doi: 10.3390/vaccines8040576 33019663 PMC7711984

[33] Dong D, Xu RH, Wong EL-Y, Hung C-T, Feng D, Feng Z, et al. Public preference for COVID-19 vaccines in China: A discrete choice experiment. Health Expect. 2020;23: 1543–1578. doi: 10.1111/hex.13140 33022806 PMC7752198

[34] Duong TV, Lin C-Y, Chen S-C, Huang Y-K, Okan O, Dadaczynski K, et al. Oxford COVID-19 Vaccine Hesitancy in School Principals: Impacts of Gender, Well-Being, and Coronavirus-Related Health Literacy. Vaccines. 2021;9: 985. doi: 10.3390/vaccines9090985 34579222 PMC8471420

[35] Costantini H. COVID-19 Vaccine Literacy of Family Carers for Their Older Parents in Japan. Healthcare (Basel). 2021;9: 1038. doi: 10.3390/healthcare9081038 34442175 PMC8393727

[36] Neumann-Böhme S, Varghese NE, Sabat I, Barros PP, Brouwer W, van Exel J, et al. Once we have it, will we use it? A European survey on willingness to be vaccinated against COVID-19. Eur J Health Econ. 2020;21: 977–982. doi: 10.1007/s10198-020-01208-6 32591957 PMC7317261

[37] Tam CC, Sun S, Yang X, Li X, Zhou Y, Shen Z. Psychological Distress Among HIV Healthcare Providers During the COVID-19 Pandemic in China: Mediating Roles of Institutional Support and Resilience. AIDS Behav. 2021;25: 9–17. doi: 10.1007/s10461-020-03068-w 33089356 PMC7577363

[38] Ye Z, Yang X, Zeng C, Wang Y, Shen Z, Li X, et al. Resilience, Social Support, and Coping as Mediators between COVID-19-related Stressful Experiences and Acute Stress Disorder among College Students in China. Applied Psychology: Health and Well-Being. 2020;12: 1074–1094. doi: 10.1111/aphw.12211 32666713 PMC7405224

[39] Finbråten HS, Pettersen KS, Wilde-Larsson B, Nordström G, Trollvik A, Guttersrud Ø. Validating the European Health Literacy Survey Questionnaire in people with type 2 diabetes: Latent trait analyses applying multidimensional Rasch modelling and confirmatory factor analysis. Journal of Advanced Nursing. 2017;73: 2730–2744. doi: 10.1111/jan.13342 28543754

[40] Bai W, Cai H, Liu S, Liu H, Qi H, Chen X, et al. Attitudes toward COVID-19 vaccines in Chinese college students. Int J Biol Sci. 2021;17: 1469–1475. doi: 10.7150/ijbs.58835 33907510 PMC8071773

[41] Barello S, Nania T, Dellafiore F, Graffigna G, Caruso R. “Vaccine hesitancy” among university students in Italy during the COVID-19 pandemic. Eur J Epidemiol. 2020;35: 781–783. doi: 10.1007/s10654-020-00670-z 32761440 PMC7409616

[42] Sharma M, Davis RE, Wilkerson AH. COVID-19 Vaccine Acceptance among College Students: A Theory-Based Analysis. Int J Environ Res Public Health. 2021;18: 4617. doi: 10.3390/ijerph18094617 33925327 PMC8123652

[43] Saied SM, Saied EM, Kabbash IA, Abdo SAE-F. Vaccine hesitancy: Beliefs and barriers associated with COVID-19 vaccination among Egyptian medical students. J Med Virol. 2021;93: 4280–4291. doi: 10.1002/jmv.26910 33644891 PMC8013865

[44] Sallam M, Dababseh D, Eid H, Hasan H, Taim D, Al-Mahzoum K, et al. Low COVID-19 Vaccine Acceptance Is Correlated with Conspiracy Beliefs among University Students in Jordan. Int J Environ Res Public Health. 2021;18: 2407. doi: 10.3390/ijerph18052407 33804558 PMC7967761

[45] Patil U, Kostareva U, Hadley M, Manganello JA, Okan O, Dadaczynski K, et al. Health Literacy, Digital Health Literacy, and COVID-19 Pandemic Attitudes and Behaviors in U.S. College Students: Implications for Interventions. Int J Environ Res Public Health. 2021;18: 3301. doi: 10.3390/ijerph18063301 33806763 PMC8004744

[46] Afonso N, Kavanagh M, Swanberg S. Improvement in attitudes toward influenza vaccination in medical students following an integrated curricular intervention. Vaccine. 2014;32: 502–506. doi: 10.1016/j.vaccine.2013.11.043 24269620

[47] Painter JE, Sales JM, Pazol K, Wingood GM, Windle M, Orenstein WA, et al. Adolescent attitudes toward influenza vaccination and vaccine uptake in a school-based influenza vaccination intervention: a mediation analysis. J Sch Health. 2011;81: 304–312. doi: 10.1111/j.1746-1561.2011.00595.x 21592125

[48] Umakanthan S, Bukelo MM, Gajula SS. The Commonwealth Caribbean COVID-19: Regions Resilient Pathway During Pandemic. Front Public Health. 2022;10: 844333. doi: 10.3389/fpubh.2022.844333 35664108 PMC9160791

[49] Jarrett C, Wilson R, O’Leary M, Eckersberger E, Larson HJ, SAGE Working Group on Vaccine Hesitancy. Strategies for addressing vaccine hesitancy—A systematic review. Vaccine. 2015;33: 4180–4190. doi: 10.1016/j.vaccine.2015.04.040 25896377

