# Supplementary material for: Perceived health literacy and COVID-19 vaccine acceptance among Chinese college students: A mediation analysis
Source: PLoS One. 2022 Sep 2;17(9):e0273285. doi: 10.1371/journal.pone.0273285 (PMC9439250; doi:10.1371/journal.pone.0273285)
Supplement: S1 Table — (DOCX) [file pone.0273285.s001.docx]

**S1 Table. Descriptive statistics and composite reliability of survey measures.**

| **Variable** | **Mean(SD) or n(%)  N=2587** | **Range** | **Cronbatch's alpha** |
| --- | --- | --- | --- |
| **Self-efficacy of COVID-19 vaccine** | 13.35 (2.68) | 4-20 | 0.820 |
| Even if nobody I know is willing to take the vaccine, I will take it | 3.5 (0.85) |  |  |
| Even there is some out-of-packet cost, I will take the vaccine | 3.56 (0.82) |  |  |
| Even there is some side effect, I will take the vaccine | 2.96 (0.84) |  |  |
| Even it needs multiple doses over time, I will take the vaccine | 3.33 (0.82) |  |  |
| **Positive attitudes towards general vaccination** | 15.85 (3.04) | 7-28 | 0.465 |
| Vaccines are safe. | 4.52 (1.44) |  |  |
| Vaccines have side effects. | 3.32 (1.16) |  |  |
| Vaccines contain dangerous ingredients. | 3.71 (1.15) |  |  |
| Vaccines cause long-term harms. | 4.3 (1.14) |  |  |
| **Perceived health Literacy** | 33.54 (4.69) | 11-44 | 0.898 |
| Understand what your doctor says to you. | 2.96 (0.59) |  |  |
| Understand the leaflets that come with your medicine. | 2.87 (0.62) |  |  |
| Understand what to do in a medical emergency. | 2.66 (0.66) |  |  |
| Understand your doctor's or pharmacist's instruction on how to take a prescribed medicine. | 2.89 (0.67) |  |  |
| Understand advice on health from family members or friends. | 3.12 (0.56) |  |  |
| Understand information on food packaging. | 3.15 (0.59) |  |  |
| Understand information in the media on how to get healthier. | 3.08 (0.58) |  |  |
| Understand information on how to keep your mind healthy. | 3.13 (0.58) |  |  |
| Understand health warnings about behaviors such as smoking, low physical activity or drinking too much. | 3.28 (0.61) |  |  |
| Understand why you need vaccinations. | 3.18 (0.59) |  |  |
| Understand why you need health screenings. | 3.23 (0.6) |  |  |
| **COVID-19 vaccine acceptance** |  | 1-5 | - |
| How likely will you get a COVID-19 vaccine when it is available? | 3.91 (0.87) |  |  |
